# Anti-cancer activity of novel dibenzo[b,f]azepine tethered isoxazoline derivatives

**DOI:** 10.1186/1472-6769-12-5

**Published:** 2012-10-03

**Authors:** Maralinganadoddi Panchegowda Sadashiva, Basappa, Shivananju NanjundaSwamy, Feng Li, Kanjoormana Aryan Manu, Murugan Sengottuvelan, Doddakunche Shivaramu Prasanna, Nirvanappa Chikkagundagal Anilkumar, Gautam Sethi, Kazuyuki Sugahara, Kanchugarakoppal Subbegowda Rangappa

**Affiliations:** 1 Department of Studies in Chemistry, University of Mysore, Mysore, 570006, India; 2 Laboratory of Proteoglycan Signalling and Therapeutics, Faculty of Advanced Life Science, Hokkaido University Graduate School of Life Science, Sapporo, 110021, Japan; 3 Department of Chemistry, Central College Campus, Bangalore University, Bangalore, 560001, India; 4 Department of Pharmacology, Yong Loo Lin School of Medicine, National University of Singapore, Kent Ridge, Singapore, 117597

**Keywords:** Dibenzoazepine, Cycloaddition, Isoxazolines, Anticancer agents, ADMET

## Abstract

**Background:**

Dibenzoazepine (DB) derivatives are important and valuable compounds in medicinal chemistry. The synthesis and chemotherapeutic properties of naturally occurring DBs and different heterocyclic moiety tethered DBs are reported. Herein, we report the DB-fused hybrid structure that containing isoxazolines (DBIs) and their anti-cancer activity, which could throw light on the structural and functional features of new molecules.

**Results and Conclusion:**

The synthesis and characterization of novel ring DB tethered isoxazoline derivatives (DBIs) were carried out. After the detailed structural characterization using 2D-NMR experiments, the compounds were identified as 5-substituted isoxazolines. The effect of newly synthesized DBIs against the invasion of murine osteosarcoma (LM8G7) cells was studied. Among the tested molecules, compound **4g** (5-[−3-(4-chlorophenyl)-4,5-dihydroisoxazol-5-yl-methyl]-5 *H-*dibenzo[b,f]azepine), was found to inhibit the invasion of LM8G7 cells strongly, when compared to other structurally related compounds. Cumulatively, the compound **4g** inhibited the invasion MDA-MB-231 cells completely at 10 μM. In addition to anti-invasion property the compound **4g** also inhibited the migration of LM8G7 and human ovarian cancer cells (OVSAHO) dose-dependently. Compound **4g** inhibited the proliferation of LM8G7, OVSAHO, human breast cancer cells (MCF-7) and human melphalan-resistant multiple myeloma (RPMI8226-LR5) cells that are comparable to cisplatin and suramin.

## Background

**Figure 1:**
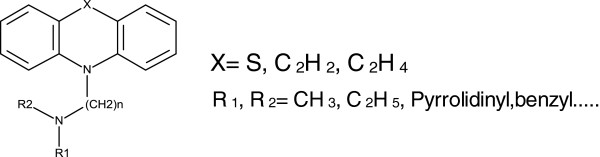
General structure of dibenzo-azepines.

Tricyclic compounds like phenothiazine, acridones, phenoxazines, benzodiazepines and dibenzoazepines are very important class of anticancer agents. Earlier studies have demonstrated that, altering the polarity (incorporation of hydrophilic or hydrophobic groups) at *N*-position play a vital role to augument the biological activity (Figure 1) [1,2]. Dibenzoazepine (DB) is a diversified core moiety which exhibit antiviral, antiepileptic, anticonvulasant, antimicrobial, antimalarial and anticancer activities [3-5]. 5 *H*-dibenzo[b,f]azepine-5-carboxamide (carbamazepine) is one of the synthesized effective anticonvulsant drugs, which is the most frequently prescribed first-line drug for the treatment of epilepsy [6]. DB-based small molecules are reported to show antioxidant activity and also sirtuin-2 inhibitory activity [7,8]. Azepine moiety containing tricyclic and naturally occurring pyrrolo[2,1-c] [1,4]benzodiazepines originated from Streptomyces species, are used as antitumor and antibiotics [9,10]. These compounds exhibit cytotoxic activity by covalent bonding between the C11-position of tricyclic azepine moiety and the N2 of the guanine residues of the minor groove of DNA [11,12]. Synthesis and anticancer activity of tricyclc azepine moiety tethered with piperazine and 1,2,3-triazole moiety are well reported [13]. Several chemical modifications of dibenzoazepine (DB) conjugated to known active moieties of heterocycles to improve the anti-cancer activity has been attempted. However they have not considered for clinical studies due to the problems relating to side effects.

Nucleosides are the building blocks of DNA and become a key molecule in the field of medicinal chemistry for the discovery of new natural nucleoside derivatives. So, extensive modifications have been performed on both the heterocyclic base as well as on the sugar moiety. Mainly, the replacement of the ribose ring with an isoxazolidine nucleus has emerged as an interesting class of dideoxynucleoside analogues [14-16]. These analogues undergo phosphorylation by cellular kinases and metabolized by enzymatic systems instead of natural nucleosides, inserted in the DNA growing chain, and finally acting as chain terminators [17,18].

In continuation of our effort to synthesise novel DBs and isoxazoli(di)ne derivatives [19-21] herein we report the synthesis of DB-fused hybrid structure that containing isoxazolines (DBIs) and their anti-cancer activity, which could throw light on the structural and functional features of new molecules.

## Results and Discussion

The dipolarophile **2** was prepared by the reaction of commercially available iminostilbene **1** with allylbromide [22]. The *N*-allyl pendant arrangement of the intermediate **2** showed a major role in the formation of product. It means that, product will be obtained on the basis of either allyl pendant bent towards the tricyclic ring or disposed outside the ring. The X-ray crystallographic studies revealed that the pendant is disposed outside from the tricyclic aromatic ring [22].

**Scheme 1:**
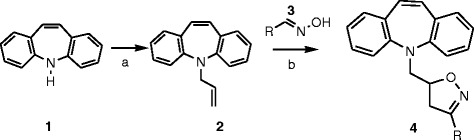
**Reagents and reaction conditions: a) 2 equiv allyl bromide, 2.5 equiv K**_**2**_**CO**_**3**_**in acetonitrile at reflux condition. b) 5 equiv oxime, 5 equiv 4% NaOCI, 1 equiv Et**_**3**_**N in MDC.**

The oxime **3** was prepared by the reaction of corresponding aldehyde with hydroxylamine in the presence of sodiumbicarbonate in EtOH/H_2_O at 60°C [19]. With two scaffolds in hand, the dibenzoazepine derivatives were synthesized in a single step operation via successive 1,3-dipolar cycloaddition reaction. A solution of oxime **3** (2 eq) in dichloromethane was added to a mixture of *N*-allyl tricyclic amine **2** (1 eq), sodium hypochlorite (4% aqueous solution, 5 eq) and Et_3_N (0.05 eq) as shown in Scheme 1.

Progress of the reaction was monitored using TLC. After completion of the reaction, the compounds **4(a-h)** were purified using column chromatography. The structures of the final products as well as intermediate were confirmed by NMR, mass and elemental analysis.

**Figure 2:**
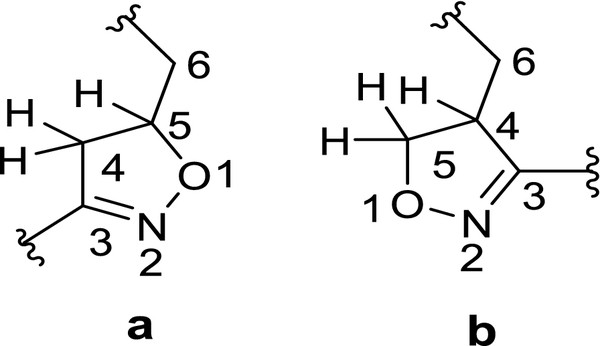
Representation of 5-substituted (a) and 4-substituted (b) isoxazoline rings.

The proton assignment and regioisomer of the title compound was studied using ^1^ H, ^13^C NMR and 2D NMR experiments (HMQC, HMBC, and COSY) (Tables 1 and 2). The chemical shift of geminal protons (H_4a_, H_4e_) and methine proton (H_5_) of isoxazoline ring were observed at 3.18, 3.36 and 4.91 ppm (^13^C; C-4; 3.18 and C-5; 78.17 ppm) respectively. The calculated chemical shifts for geminal protons 4-substituted isoxazoline was assigned at ~5.0 ppm due to the neighboring O-atom effect [23] (Figure 2). Further, HMBC experiment also gave the significant information for the regioselectivity. The geminal protons H_4a_, H_4e_ showing the cross peak with C-3 (−C = N-) of isoxazoline ring. Similarly, C-5 signal gave cross peak with geminal protons H_4a_, H_4e_ but not with C-3 (−C = N-) (Table 1). The above data strongly suggested that, the obtained product is 5-substituted isoxazoline.

**Table 1:** ^**1**^**H and **^**13**^**C NMR (HMQC and HMBC) data of the compound, 5-[-3-(4-Chlorophenyl)-4,5-dihydroisoxazol-5-ylmethyl]-5*H *dibenzo[b,f]azepine**

	**HMBC**					**HMQC**			
	**Carbon**	**C**_**4**_	**C**_**6**_	**C**_**5**_		**Carbon**	**C**_**4**_	**C**_**6**_	**C**_**5**_
Proton	δ ppm	38.35	53.7	78.7	Proton	δ ppm	38.35	53.7	78.7
**H**_**4a**_	3.153	Bonded	β	α	**H**_**4ax**_	3.153	Bonded		
**H**_**4b**_	3.20	Bonded	β	α	**H**_**4eq**_	3.20	Bonded		
**H**_**6a**_	3.483	β	Bonded	α	**H**_**6a**_	3.483		Bonded	
**H**_**6b**_	4.295	β	Bonded	α	**H**_**6b**_	4.295		Bonded	
**H**_**5**_	4.886	α	β	Bonded	**H**_**5**_	4.886			Bonded

α= ^2^J(C,H).

β= ^3^J(C,H).

**Table 2:** **2D-NMR data (COSY) of the compound, 5-[-3-(4-chlorophenyl)-4, 5-dihydroisoxazol-5-yl methyl]-5*H *dibenzo [b,f]azepine**

	**H-H-COSY**		^**1**^**H NMR**	^**13**^**C NMR**
**Position**	**Chemical Shifts in ppm**	**Position**	**δ ppm**^**1**^**H**	**δ ppm**^**13**^**C**
**H**_**4a**_	3.153/3.208			
3.153/4.886	**H**_**4ax**_**(C-4)**	3.18	38.15	
**H**_**4b**_	3.208/3.153			
3.208/4.886	**H**_**4eq**_**(C-4)**	3.36	38.15	
**H**_**6a**_	3.483/4.295			
3.483/4.886	**H**_**6a**_**(C-6)**	3.59	53.7	
**H**_**6b**_	4.295/3.483			
4.295/4.886	**H**_**6b**_**(C-6)**	4.31	53.7	
**H**_**5**_	4.886/3.153			
4.886/3.208				
4.886/3.483				
4.886/4.296	**H**_**5**_**(C-5)**	4.91	78.7	
		**C-3**		155.56
		**Ar-H**	6.5-7.7	120-136

## Biology

### DBIs inhibited the invasion of murine osteosarcoma and human breast cancer cells

**Figure 3:**
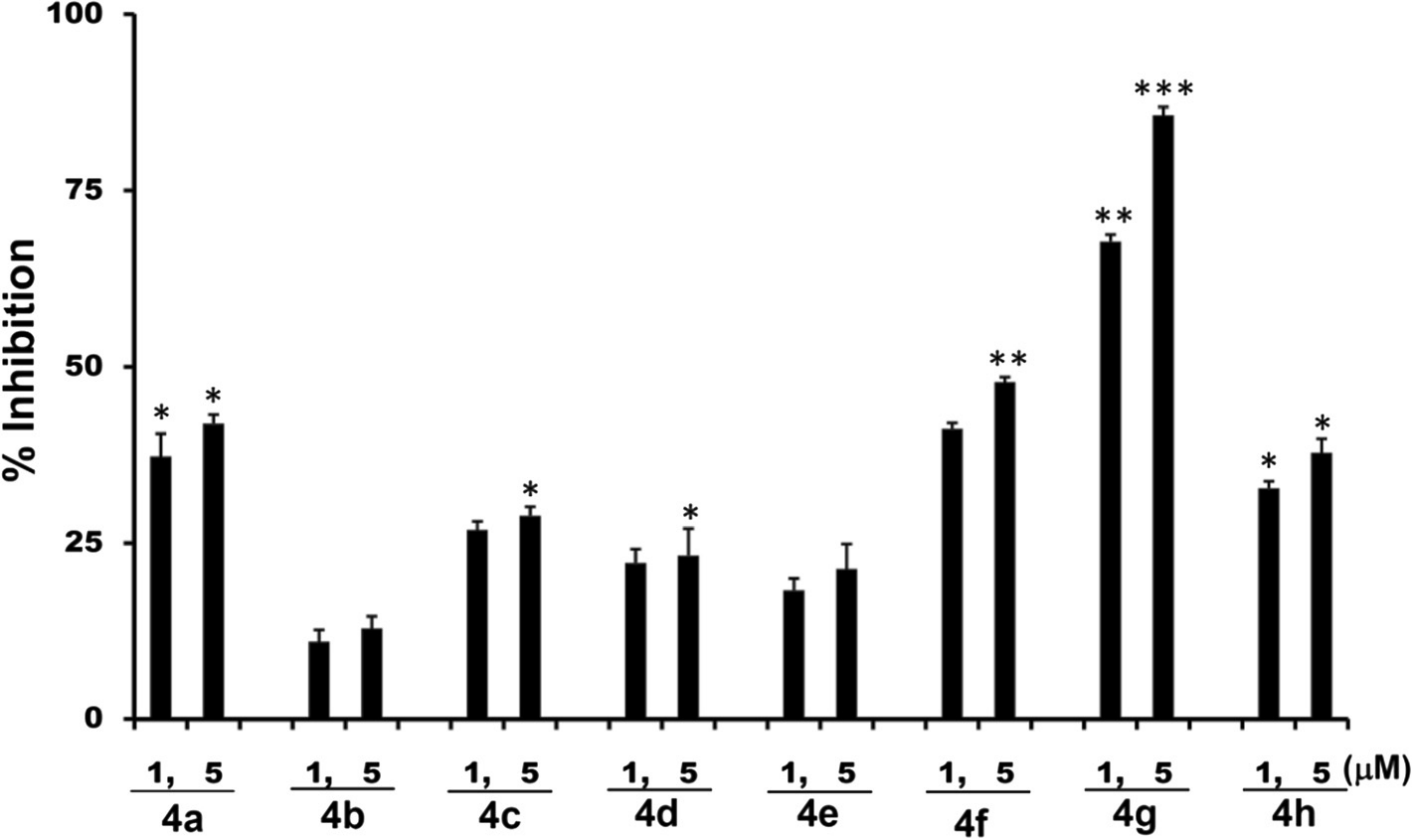
**DBIs inhibited the invasion of LM8G7 cells.** LM8G7 cells were seeded in Boyden-chambers in triplicate and incubated for 24 h with DMEM or a medium containing 1 or 5 μM of DBIs derivatives. The cell invasion and its inhibition by DBIs were measured. The % inhibition of the invasion of LM8G7 cells by DBIs was given. The data represent the mean value ± S.D. for three independent experiments. ^*^P < 0.05 versus control, **p 0.01 versus control, ***p 0.001 versus control, Mann–Whitney U test.

**Figure 4:**
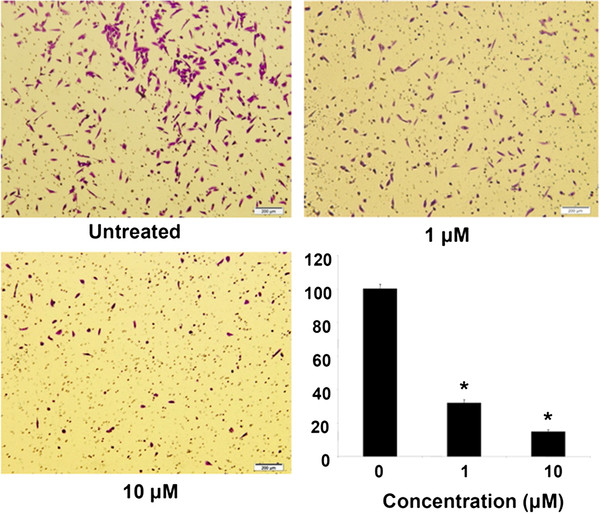
**Compound 4g completely inhibited the invasion of MDA-MB-231 cells.** Cells were seeded in Matrigel^TM^-coated Boyden chambers and were incubated for 24 h with DMEM (untreated) or a medium containing 1 μM of compound **4g** (1 μM) or 10 μM compound **4g** (10 μM); the photographs shows the cell on the lower surface of the filter (invaded) stained with the Diff-Quick solution. The % inhibition of the invasion of MDA-MB-231 by compound **4g** was presented. The data represent the mean value ± S.D. for three independent experiments. ^*^ P < 0.05 versus control, Student's t-test.

Invasion and metastasis are the life-threatening aspects of cancer cells [24]. Most of the cancers unmask their invasive property, and thereby progressing to frank malignancy from pre-existing carcinoma *in situ*, or from disorders of epithelial proliferation. Hence, we initially studied the effects of DBIs on the invasion of highly metastatic murine osteosarcoma (LM8G7) cells using Matrigel^TM^-coated porous membranes. The LM8G7 cells were highly invasive in the assay. The efficacy of the compounds against the invasion of LM8G7 was summarized. Among the tested DBIs, **4g** at 1 and 5 μM concentration inhibited the invasion of LM8G7 cells by 67 and 85%, respectively. The results indicated that the compound **4g** exhibited dose-dependent anti-invasive property against the LM8G7 cells, when compared to the other structurally similar derivatives (Figure 3). Suramin inhibited the invasion of LM8G7 cells by 73 and 94% at 1 and 5 μM, respectively (data not shown). Furthermore, we selected the compound **4g** and evaluated its effect on the invasion of human breast cancer cells (MDA-MB-231) *in vitro*. The results of the study revealed that the compound **4g** completely suppressed the invasion of MDA-MB-231 cells in a dose-dependent manner (Figure 4).

### Compound 4g inhibited the migration of tumor cells

**Figure 5:**
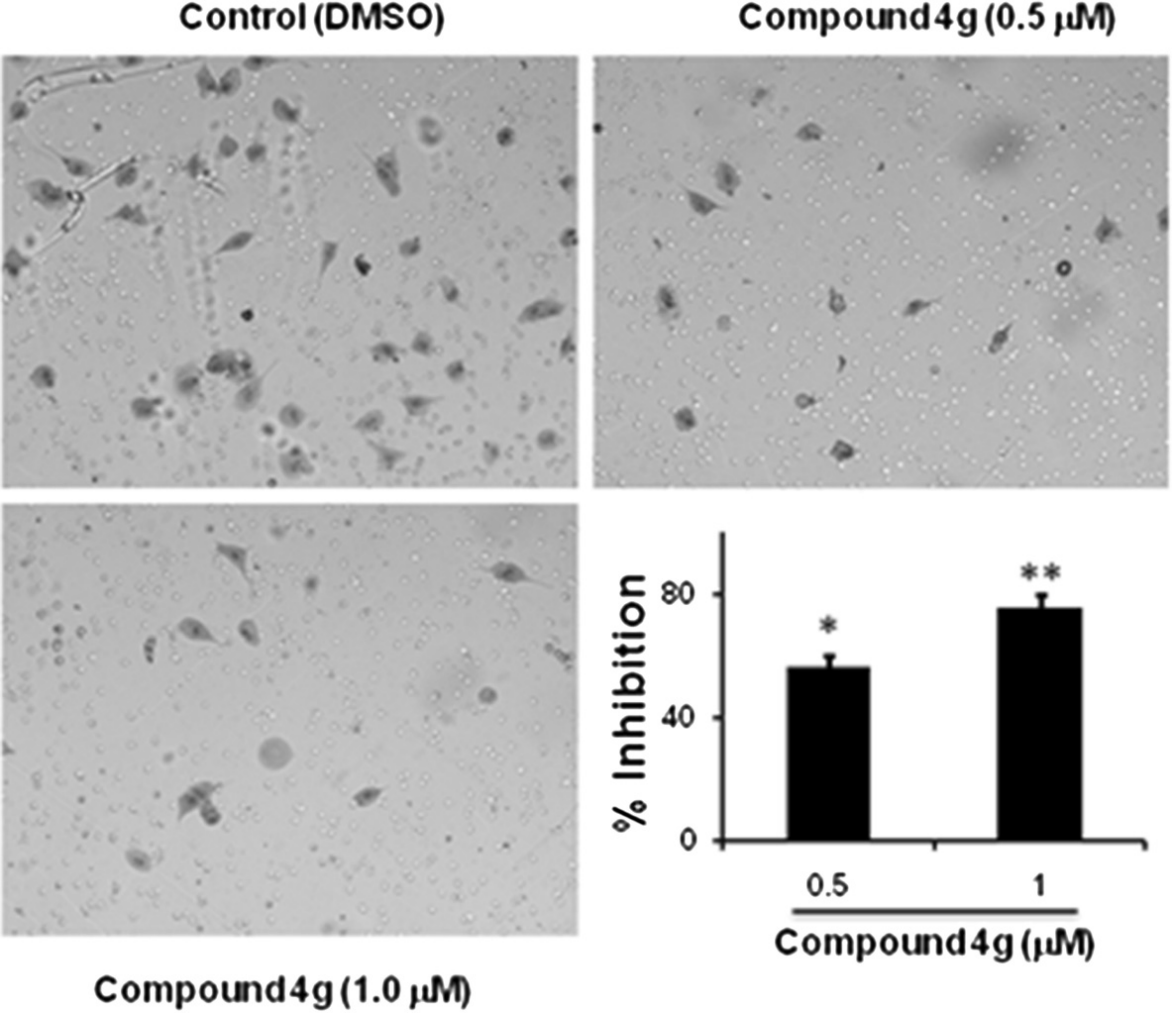
**Compound 4g inhibited the migration of LM8G7 cells.** Cells were seeded in control-Boyden chambers and were incubated for 24 h with DMEM or a medium containing 0.5 or 1 μM of compound **4g**; the photographs shows the cell on the lower surface of the filter (migrated) stained with Diff-Quick solution. The % inhibition of the migration of LM8G7 by compound **4g** was presented. The data represent the mean value ± S.D. for two independent experiments. ^*^ P < 0.05 versus control, ** *p* 0.01 *versus* control, Mann–Whitney *U* test.

Metastatic tumor cells can migrate from one place to another in the body. So, the effect of compound **4g** against the migration of LM8G7 cells was studied. Boydon Chamber assay was performed to examine its effect on migration. Compound **4g** inhibited the migration of LM8G7 cells dose dependently (Figure 5) at 0.5 and 1 μM by 47 and 78% respectively. Furthermore, the compound **4g** also inhibited the migration of human ovarian (OVSAHO) cells by 40.2 and 84.9% at 0.5 and 1.0 μM, respectively (data not shown).

### Compound 4g inhibited the proliferation of tumor cells

**Figure 6:**
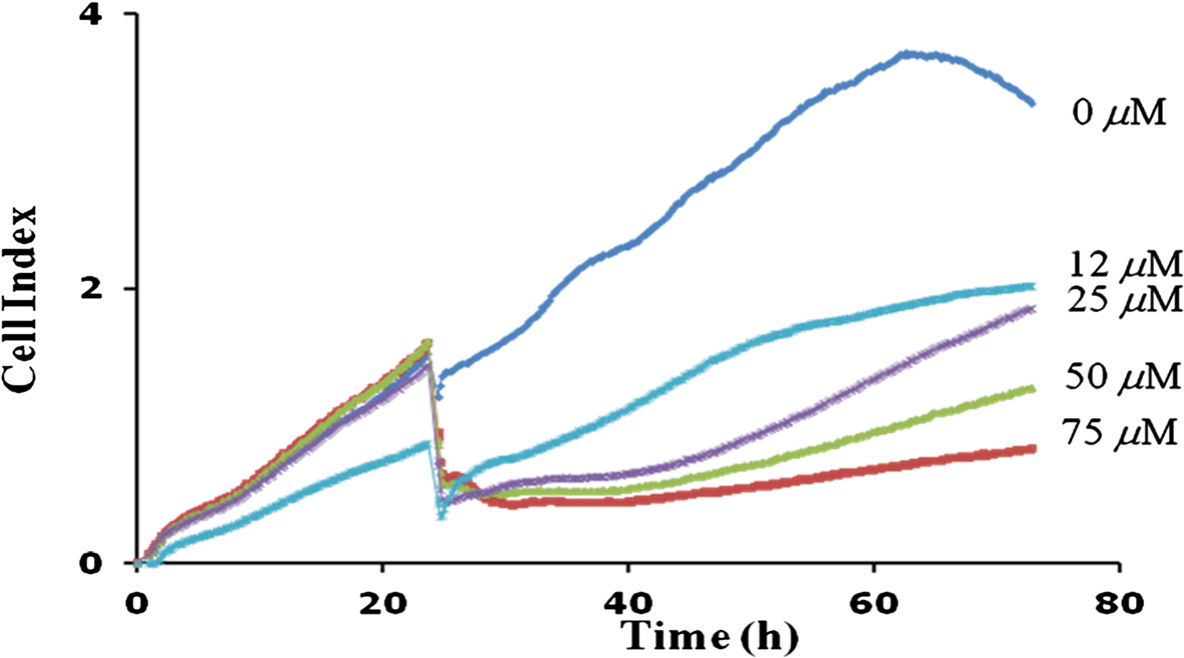
**Real-time monitoring of the effects of compound 4g on the proliferation of LM8G7 cells.** LM8G7cells were seeded in ACEA’s 96X e-plate^TM^ at a density of 5 x 10^3^ cells per well in triplicate, and continuously monitored using the RT-CES system up to 24 h, at which point compound **4g** (12 to 75 μM) was added. The cell index is plotted against the time. Data represent the mean values ± S.D. for three identical wells from three independent experiments. *p 0.05 *versus* control, Mann–Whitney *U* test.

**Figure 7:**
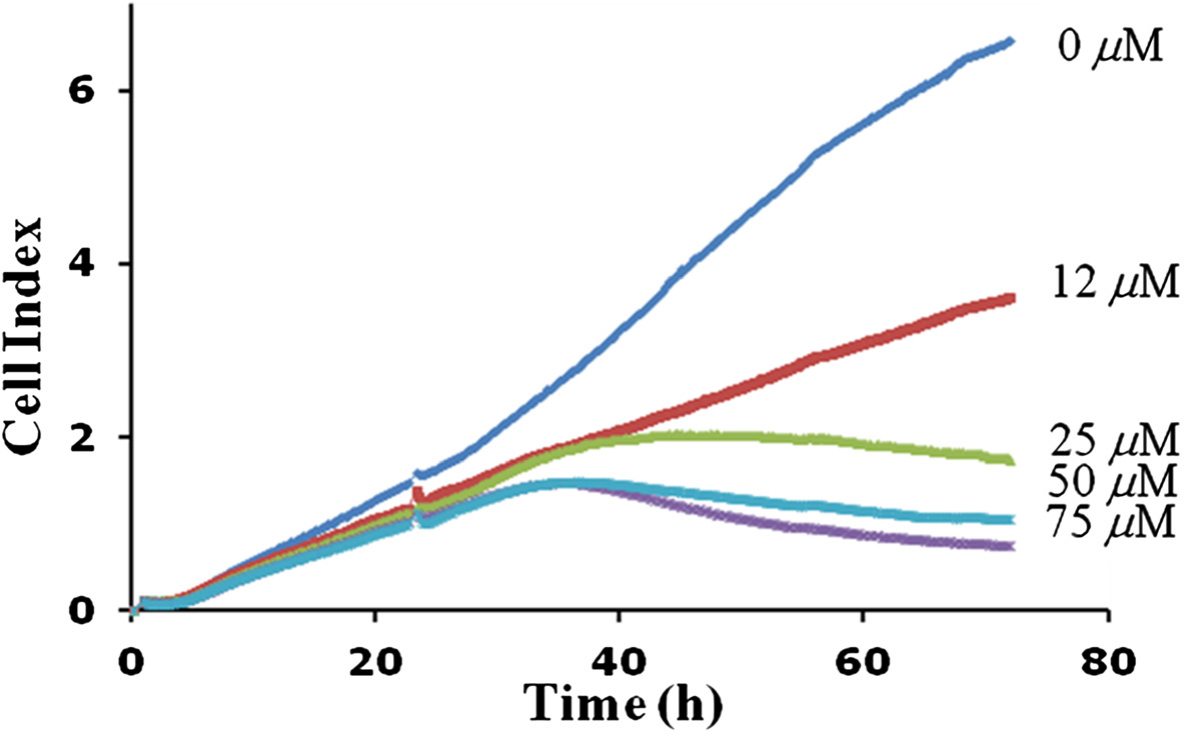
**Real-time monitoring of the effects of compound 4g on the proliferation of OVSAHO cells.** OVSAHO cells were seeded in ACEA’s 96X e-plate^TM^ at a density of 5 x 10^3^ cells per well and continuously monitored using the RT-CES system up to 24 h, at which point compound **4g** was added. The cell index is plotted against the time. Data represent the mean values ± S.D. for three identical wells from three independent experiments. *p 0.05 *versus* control, **p 0.01 *versus* control, Mann–Whitney *U* test.

**Figure 8:**
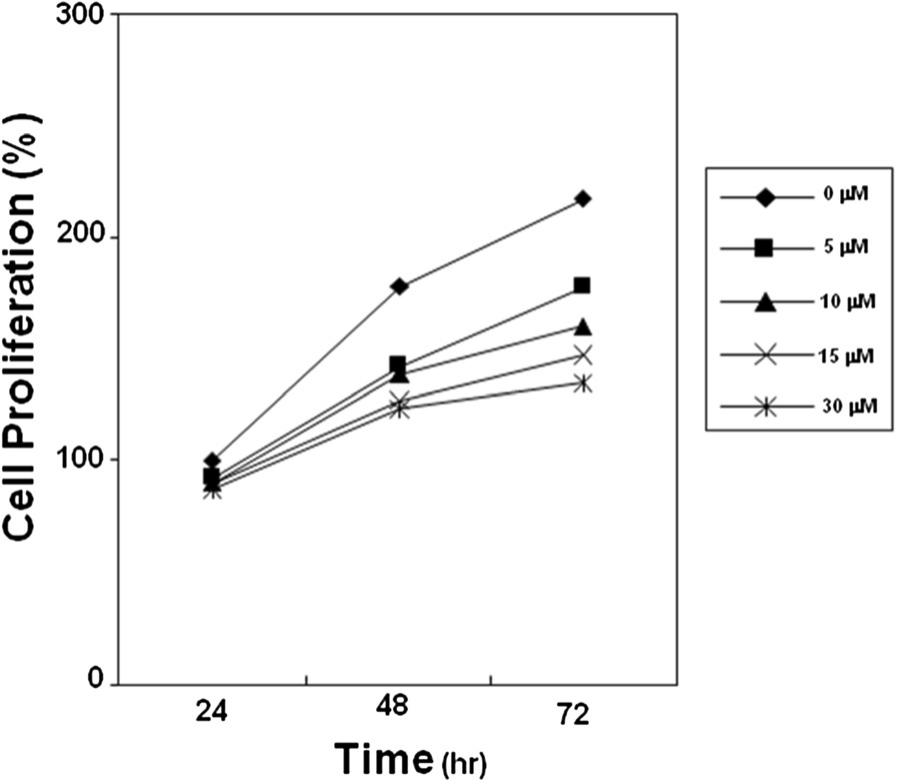
**Anti-proliferative effects of compound 4g on RPMI8226-LR5 cells.** Cells (20 x 10^3^/100 μl) were plated in triplicate, treated with 0, 5, 10, 15, and 30 μM compound **4g**, and then subjected to MTT assay on days 1, 2, and 3, to analyze proliferation of cells. Data represent the mean values ± S.D.

**Figure 9:**
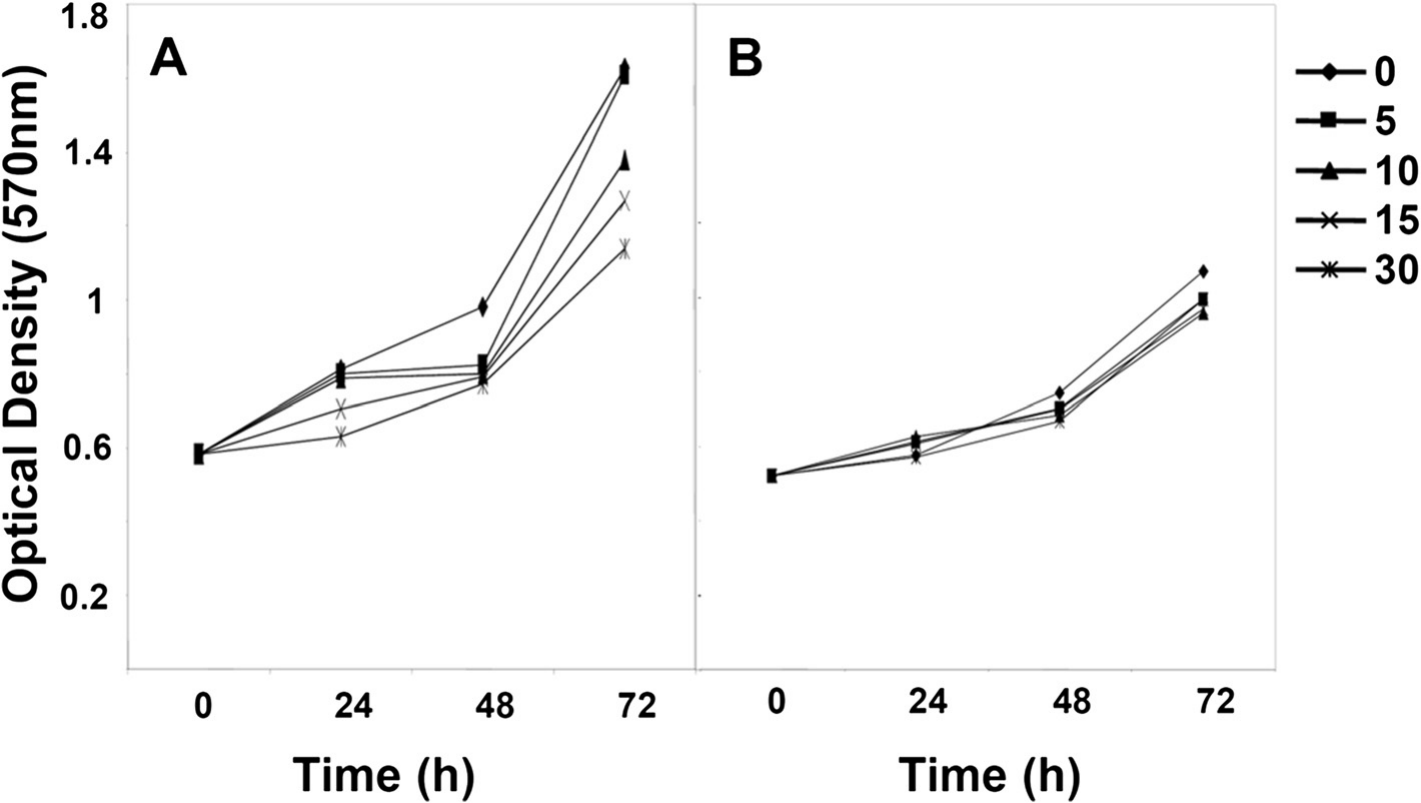
**Anti-proliferative effects of compound 4g on MCF-7 or MCF-10A cells.** MCF-7 or MCF-10A cells were plated in triplicate, treated with 0, 5, 10, 15, and 30 μM of compound **4g**, and then subjected to MTT assay on days 1, 2, and 3, to analyze proliferation of cells. *p 0.05 *versus* control (15 μM, 72 h), **p 0.01 *versus* control (30 μM, 72 h), Mann–Whitney *U* test.

We monitored the effects of compound **4g** on the proliferation of LM8G7 or OVSAHO cells using real-time cell electronic sensing system^TM^ (RT-CES). The effects of compound **4g** on the proliferation of LM8G7 or OVSAHO cells were monitored dynamically for every 10 min. Compound **4g** inhibited the proliferation of LM8G7 and OVSAHO cells dose-dependently with an IC_50_ value of 15 μM and 24 μM, respectively proved its anti-proliferative effect on tumor cells (Figure 6 and 7). We next investigated the anti-proliferative effect of compound **4g** on human melphalan-resistant multiple myeloma (RPMI8226/LR5) cells using MTT assay. It is observed that the compound **4g** exerts anti-proliferative affects on RPMI8226/LR5 at the tested concentration (Figure 8). Furthermore, we investigated the anti-proliferative effect of the compound **4g** on human breast cancer (MCF-7) cells. As shown in Figure 9a, it was observed that the compound **4g** exerts anti-proliferative effects on MCF-7 cells at various tested concentrations when compared to the control group. Interestingly, we observed that compound **4g** had little or no anti-proliferative effect on MCF-10A cells, indicating that it is not substantially cytotoxic to normal cells (Figure 9b). On the other hand, compound **4g** moderately inhibited the proliferation of mouse endothelial cells (UV♀2) with an IC_50_ value of 62 μM (Table 3). These results show that compound **4g** suppressed the proliferation of endothelial cells, but the concentration of compound **4g** required to suppress cell proliferation is high. The inhibitory activity of compound **4g** is comparable to that of reference molecules such as cisplatin and suramin.

**Table 3:** Inhibition of the proliferation of tumor and endothelial cells by compound 4g

**Compound**	**Anti-proliferative activity (IC**_**50**_**in ***μ***M)**		
	**LM8G7**	**OVSAHO**	**UV**♀**2**
**4g**	15± 0.6*	24± 3.6**	62± 1.9
Cisplatin	30 ± 24	15 ± 0.6	12 ± 0.4
Suramin	12 ± 2.8	14 ± 24	34 ± 1.4

*p 0.05 *versus* control, **p 0.01 *versus* control, Mann-Whitney *U* test.

### Compound 4g can induce apoptosis in tumor cells

**Figure 10:**
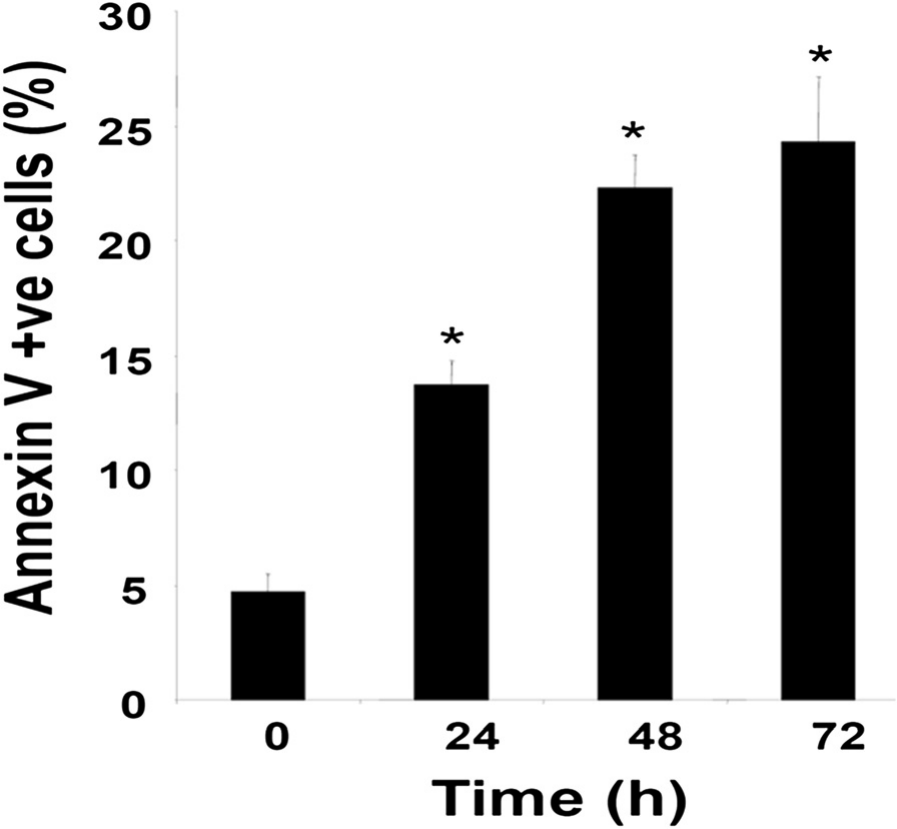
**Compound 4g induces apoptosis in MCF-7 cells.** Cells were treated with 30 μM compound **4g** for indicated times, incubated with anti-annexin V antibody conjugated with FITC, and analyzed with a flow cytometer for apoptotic effects. **p 0.05 *versus* control, Student's t-test.

We evaluated the effect of compound **4g** to induce apoptosis in tumor cells to detect early stage of apoptosis. The results indicate that the compound **4g** can induce significant apoptosis in a time dependent manner in MCF-7 cells (Figure 10).

### Absorption–distribution–metabolism–excretion–toxicity (ADMET) properties of DBIs

*In silico* ADMET properties for all the newly synthesized compounds were obtained using Discovery Studio programme (Accelrys Inc., USA). All the DBIs are in accordance with the parameters of the Lipinski’s Rule of Five [25]. The absorption (PSA2D) parameter range was 23 to 66 and also the distribution (AlogP) parameters range lies between 4.6 to 5.9 (Table 4). The ADMET-human intestinal absorption model predicts that these compounds could well absorb in the body. Probably, these compounds are highly penetrable to the blood brain barriers (BBB) after oral administration. Also, the recursive partitioning/classification trees method predicts that the compound can inhibit the CYP2D6 enzyme weakly. These pharmacokinetic parameters well within the acceptable range defined for human use, thereby indicating their potential as drug-like or drug seed molecules.

**Table 4:** ADMET-properties of the sugar mimetic isoxazoline molecules by use of Discovery Studio 2.5 version

**Compounds**	**BBB**	**Solubility**	**Hepatotoxicity**	**CYP2D6**	**PPB level**	**ADMET_AlogP98**	**ADMET_PSA_2D**
**4a**	0.42	-6.73	0.96	0.90	2	5.28	66.42
**4b**	0.42	-6.70	0.94	0.84	2	5.28	66.42
**4c**	0.69	-6.49	0.80	0.83	2	5.33	50.39
**4d**	0.99	-6.64	0.93	0.92	2	5.37	32.53
**4e**	0.73	-6.13	0.92	0.92	2	4.66	34.86
**4f**	1.13	-6.76	0.95	0.96	2	5.38	23.60
**4g**	1.34	-7.36	0.91	0.85	2	5.98	23.60
**4h**	1.26	-7.20	0.96	0.75	2	5.79	23.60

## Conclusions

In conclusion, we herein report the incorporation of isoxazoline ring tethered to dibenzo[b,f]azepine for the first time. After the detailed structural characterization using 2D-NMR experiments, the products were confirmed as 5-substituted isoxazolines. Among the tested compounds, compound **4g** was found to inhibit the invasion of LM8G7 cells, when compared to other structurally related DBIs. Also, the compound **4g** inhibited the invasion MDA-MB-231 cells completely at 10 μM. Evident to invasion, the compound **4g** also inhibited the migration of LM8G7 and OVSAHO cells dose dependently. As a result, inhibitory activity of compound **4g** on proliferation of LM8G7, OVSAHO, MCF-7 and RPMI8226/LR5 cells and was comparable to that of cisplatin and suramin.

## Methods

### Chemical synthesis and reagents

Melting points were determined in capillaries on a Tottoli apparatus and are uncorrected. The NMR experiments ^1^ H, ^13^C, HMBC, HMQC were carried out at 500 (125) MHz and the reported chemical shifts (δ) are given in ppm and the coupling constants (*J*) in Hertz (Hz). Multiplicities of NMR signals are designed as s (singlet), d (doublet), m (multiplet, for unresolved lines). Mass spectra were recorded on a Trio 1000 Thermo Quest spectrometer in the electron impact mode or a Platform Micromass spectrometer in the electro spray mode. TLC was performed on silica gel Alugram SilG/UV254 (Macherey-Nagel). The murine osteosarcoma cell line LM8G7, a highly metastatic murine osteosarcoma cell line with the potential to form tumor nodules in the liver, was cloned from LM8G5 cells as described [26,27] and cultured in DMEM supplemented with 10% FBS, streptomycin (100 μg/ml), penicillin (100 units/ml), 100X non-essential amino acids, β-mercaptoethanol (50 μM), 100X sodiumpyruvate, and L-glutamine (2 mM) at 37°C in a humidified 5% CO_2_ atmosphere. Human ovarian cancer cells (OVSAHO) were procured from ATCC and cultured in RPMI media supplemented with 10% FBS, L-glutamine (2 mM), and NaHCO3 (10%). The cells were grown to 80 % confluency before passage, and experiments were restricted to passages 5–20. The human multiple myeloma (MM) cell line RPMI-8226-LR-5 cells were cultured in RPMI 1640 medium containing 1x antibiotic-antimycotic with 10% FBS. RPMI-8226-LR-5 and MCF-7 cells were cultured in RPMI 1640 medium with 10% FBS supplemented with 100 U/mL penicillin and 100 μg/mL streptomycin. MCF-10A cells were cultured in MEGM® mammary epithelial cell complete medium obtained from Lonza, USA [28].

### Synthesis of allyl-5 H dibenzo[b,f]azepine 2

The compound **2** was synthesized using the reported method [22].

The product is yellow solid. mp: 40–42°C. ^1^ H NMR (*δ* ppm, CDCl_3_, 500 MHz): *δ* 4.4 (d, 2 H *J* = 14 Hz), 5.12 (dd, 2 H), 5.28 (dd, 2 H), 5.8 (m, 1 H), 6.76 (s, 2 H), 7.08 (d, 2 H *J* = 5 Hz), 6.98–7.12 (q, 4 H), 7.2–7.3 (t, 2 H).IR KBr (cm^-1^): 1315, 1642, 3040, 3072. Anal. Calcd for C_17_H_15_N: C, 87.6; H, 6.49; N, 6.0. Found: C, 87.63; H, 6.43; N, 6.0.

#### General procedure for the DBIs

The weighed amount of aldoxime **3** (2 eq) was dissolved in 10 mL of methylene dichloride taken in a round bottom flask. The solution was made basic using triethylamine (0.05 eq). 2 equivalents of sodiumhypochlorite, followed by tricyclicamine (1 equiv) **2,** were added slowly at 0°C to the stirring solution. After complete addition of the reactants stirring was continued until the reaction is completed. The crude residue was purified by silica gel column chromatography eluting with a mixture of n-hexane and ethyl acetate with successive increasing the quantity of ethyl acetate, affording the corresponding product **4(a-h)**.

##### 5-[−3-(2-nitrophenyl)-4,5-dihydroisoxazol-5-yl-methyl]-5 H-dibenzo[b,f] azepine 4a

The product is a thick liquid. Yield: 0.229 g (67.3%).^1^ H NMR (*δ* ppm, CDCl_3_, 500 MHz): δ 3.28 (dd, 1 H, H_4a_, *J*-14.4, 7.5 Hz); 3.37 (dd, 1 H, H_4e_, *J*-14.4, 5.2 Hz); 3.51 (dd, 1 H, H_6a_, *J*-12.1, 7.8 Hz); 4.33 (dd, 1 H, H_6e_, *J*-12.1, 4.1 Hz); 4.88 (m, 1 H, H_5_); 6.76 (d, 2 H, CH, *J*-2.1 Hz); 7.1-8.20 ( m, 12 H, Ar-H). ^13^C NMR (*δ* ppm, CDCl_3_, 125 MHz): δ 38.2 (C-4), 55.6 (C-6), 77.5 (C-5), 124 (CH), 127–144.0 (Ar-C), 158.2 (C-C = N). MS (ESI + ion): m/z = 398.1 [M + H] ^+^. Anal. calcd for C_24_ H_19_ N_3_O_3_: C, 72.53; H, 4.82; N, 10.57. Found : C, 72.45; H, 4.86; N, 10.48.

#### 5-[−3-(3-nitrophenyl)-4, 5-dihydroisoxazol-5-yl-methyl]-5 H-dibenzo[b,f]azepine 4b

The product is a thick liquid. Yield: 0.224g (65.7 %). ^1^ H NMR (*δ* ppm, CDCl_3_, 500 MHz): δ 3.24 (dd, 1 H, H_4a_, *J*-14.1, 7.2 Hz); 3.37 (dd, 1 H, H_4e_, *J*-14.1, 5.0 Hz); 3.45 (dd, 1 H, H_6a_, *J*-12.8, 6.8 Hz); 4.33 (dd, 1 H, H_6e_, *J*-12.8, 4.6 Hz); 4.77 (m, 1 H, H_5_); 6.70 (d, 2 H, CH, *J*-2.1 Hz); 7.4-8.26 (m, 12 H, Ar-H). ^13^C NMR (*δ* ppm, CDCl_3_, 125 MHz): δ 38.6 (C-4), 54.3 (C-6), 76.4 (C-5), 125.2 (CH), 130–146.2 (Ar-C), 154.8 (C-C = N). MS (ESI + ion): m/z = 398.6 [M + H] ^+^. Anal. calcd for C_24_ H19 N_3_O_3_: C, 72.53; H, 4.82; N, 10.57. Found : C, 72.48; H, 4.78; N, 10.41.

##### 5-[−3-(3,4,5-trimethoxyphenyl)-4,5-dihydroisoxazol-5-yl-methyl]5 H-dibezo[b,f] azepine 4c

The product is a thick liquid. Yield: 0.260 g (68.6%). ^1^ H NMR (*δ* ppm, CDCl_3_, 500 MHz): δ 3.21 (dd, 1 H, H_4a_, *J*-15.8, 9.6 Hz); 3.30 (dd, 1 H, H_4e_, *J*-15.8, 6.1 Hz); 3.48 (dd, 1 H, H_6a_, *J*-12.5, 8.1 Hz); 3.76 (s, 6 H, OCH_3_); 3.79(s, 3 H, OCH_3_); 4.28 (dd, 1 H, H_6e_, *J*-13.1, 4.4 Hz); 4.84 (m, 1 H, H_5_); 6.78 (d, 2 H, CH, *J*-2.2 Hz); 6.82-7.42 (m, 10 H, Ar-H). ^13^C NMR (*δ* ppm, CDCl_3_, 125 MHz): δ 37.5 (C-4), 54.7 (C-6), 56.4 (OCH_3_), 76.9 (C-5), 126 (CH), 128–134.0 (Ar-C), 156.6 (C-C = N). MS (ESI + ion): m/z = 443.5 [M + H] ^+^. Anal. calcd for C_27_ H_26_ N_2_O_4_: C, 73.2; H, 5.92; N, 6.33. Found : C, 73.15; H, 5.86; N, 6.28.

##### 5-[−3-(4-methoxyphenyl)-4,5-dihydroisoxazol-5-yl-methyl]-5 H-dibenzo[b,f] azepine 4d

The product is a thick liquid. Yield: 0.214g (65.3 %). ^1^ H NMR (*δ* ppm, CDCl_3_, 500 MHz): δ 3.24 (dd, 1 H, H_4a_, *J*-16.8, 9.8 Hz); 3.36 (dd, 1 H, H_4e_, *J*-16.8, 6.8 Hz); 3.50 (dd, 1 H, H_6a_, *J*-14.8, 8.8 Hz); 3.72 (s, 3 H, OCH_3_); 4.30 (dd, 1 H, H_6e_, *J*-14.8, 4.5 Hz); 4.88 (m, 1 H, H_5_); 6.64 (d, 2 H, CH, *J*-2.8 Hz); 6.82-7.32 ( m, 12 H, Ar-H). ^13^C NMR (*δ* ppm, CDCl_3_, 125 MHz): δ 36.2 (C-4), 53.8 (C-6), 56.8 (OCH_3_), 76.2 (C-5), 126.8 (CH), 128–136.0 (Ar-C), 158.1 (C-C\= N). MS (ESI + ion): m/z = 383.75 [M + H] ^+^. Anal. calcd for C_25_ H_22_ N_2_O_2_: C, 78.51; H, 5.80; N, 7.32. Found : C, 78.58; H, 5.89; N, 5.68.

#### Synthesis of 5-[−3-(pyridyl)-4,5-dihydroisoxazol-5-yl-methyl]-5 H-dibenzo [b,f] azepine 4e

The product is thick liquid. Yield: 0.22 g (72.6 %). ^1^ H NMR (*δ* ppm, CDCl_3_, 500 MHz): δ 2.88 (dd, 1 H, H_4a_, *J*-16.7, 10.7 Hz); 3.28 (dd, 1 H, H_4e_, *J*-16.5, 6.0 Hz); 3.39 (dd, 1 H, H_6a_, *J*-13.0, 9.0 Hz); 4.27 (dd, 1 H, H_6e_, *J*-12.5, 4.5 Hz); 4.72 (m, 1 H, H_5_); 6.70 (d, 2 H, CH, *J*-2.5 Hz); 6.52-8.22 (m, 12 H, Ar-H). ^13^C NMR: δ 36.4 (C-4), 51.8 (C-6), 75.51 (C-5), 124.22 (CH), 124–152.9 (Ar-C), 158.21 (C-C = N). MS (ESI + ion): m/z =354.0 [M + H] ^+^. Anal. calcd for C_23_ H_19_ N_3_O: C, 78.16; H, 5.42; N, 11.89. Found : C, 78.21; H, 5.34; N, 11.78.

##### 5-[−3-(phenyl)-4,5-dihydroisoxazol-5-yl-methyl]-5 H-dibenzo[b,f]azepine 4(f

The product is yellow thick liquid. Yield: 0.23 g (72.3 %). ^1^ H NMR (*δ* ppm, CDCl_3_, 500 MHz): δ 3.11 (dd, 1 H, H_4a_, *J*-15.0, 10.2 Hz); 3.28 (dd, 1 H, H_4e_, *J*-15.0, 5.4 Hz); 3.48 (dd, 1 H, H_6a_, *J*-12.5, 8.5 Hz); 4.28 (dd, 1 H, H_6e_, *J*-12.3, 4.1 Hz); 4.84 (m, 1 H, H_5_); 6.73 (d, 2 H, CH, *J*-2.5 Hz); 7.00-7.33 (m, 13 H, Ar-H). ^13^C NMR (*δ* ppm, CDCl_3_, 125 MHz): δ 36.10 (C-4), 51.25 (C-6), 75.81 (C-5), 121 (CH), 122–131.0 (Ar-C), 153.00 (C-C = N). MS (ESI + ion): m/z =353.1 [M + H] ^+^. Anal. calcd for C_24_ H_19_ N_2_FO: C, 77.82; H, 5.17; N, 7.56. Found : C, 77.9; H, 5.21; N, 7.48.

##### 5-[-3-(4-chlorophenyl)-4,5-dihydroisoxazol-5-yl-methyl]-5 H-dibenzo[b,f] azepine 4g

The product is yellow solid. Yield: 0.25 g (75 %). mp-156–158°C. ^1^ H NMR (*δ* ppm, CDCl_3_, 500 MHz)**:** δ 3.15 (dd, 1 H, H_4a_, *J*-16.7, 10.7 Hz); 3.20 (dd, 1 H, H_4e_, *J*-16.5, 6.0 Hz); 3.45 (dd, 1 H, H_6a_, *J*-13.0, 9.0 Hz); 4.30 (dd, 1 H, H_6e_, *J*-12.5, 4.5 Hz); 4.29 (m, 1 H, H_5_); 6.73 (d, 2 H, CH, *J*-2.5 Hz); 7.00-7.52 (m, 12 H, Ar-H). ^13^C NMR (*δ* ppm, CDCl_3_, 125 MHz): δ 38.54 (C-4), 53.78 (C-6), 78.71 (C-5), 124 (CH), 127–135.9 (Ar-C), 155.56 (C-C = N). MS (ESI + ion): m/z =387.0 [M + H] ^+^. Anal. calcd for C_24_ H_19_ N_2_ClO: C, 74.51; H, 4.95; N, 7.24. Found : C, 75.08; H, 5.14; N, 7.08.

#### 5-[-3-(2,6-difluorophenyl)-4,5-dihydroisoxazol-5-yl-methyl]-5 H-dibenzo[b,f] azepine 4h

The product is thick liquid. Yield: 0.209 g (69.2 %). ^1^ H NMR (*δ* ppm, CDCl_3_, 500 MHz): δ 3.11 (dd, 1 H, H_4a_, *J*-14.7, 9.8 Hz); 3.24 (dd, 1 H, H_4e_, *J*-14.7, 5.5 Hz); 3.48 (dd, 1 H, H_6a_, *J*-12.0, 7.0 Hz); 4.31 (dd, 1 H, H_6e_, *J*-12.0, 3.8 Hz); 4.71 (m, 1 H, H_5_); 6.68 (d, 2 H, CH, *J*-2.2 Hz); 6.8-7.62 (m, 11 H, Ar-H). ^13^C NMR (*δ* ppm, CDCl_3_, 125 MHz): δ 34.70 (C-4), 51.80 (C-6), 76.61 (C-5), 123.75 (CH), 128.8-165.9 (Ar-C), 158.80 (C-C = N). MS (ESI + ion): m/z =389.14 [M + H] ^+^. Anal. calcd for C_24_ H_18_ N_2_F_2_O: C, 74.21; H, 4.67; N, 7.21. Found : C, 74.11; H, 4.54; N, 7.10.

### Invasion assay

The *in vitro* invasion assay was performed using bio-coat Matrigel invasion assay system (BD Biosciences, San Jose, CA, USA), according to the manufacturer’s instructions. MDA-MB-231 cells (2 × 10^5^ cells) or LM8G7 cells (2.5 x 10^4^) were suspended in serum-free RPMI 1640 medium or DMEM, respectively and seeded into the Matrigel transwell chambers consisting of polycarbonate membranes with 8-μm pores [29]. After incubation with 1 or 10 μM concentrations of DBIs for 24 h, the upper surfaces of the transwell chambers were wiped with cotton swabs and the invading cells were fixed and stained with crystal violet solution. The invading cell numbers were counted in five randomly selected microscope fields. The % inhibition of the invaded cells was calculated.

### In vitro migration assay

The efficacy of LM8G7 cell migration was assessed using the BD BioCoat^TM^ chamber (8 μm PET pores) without Matrigel (BD Biosciences, 8 μm pore size, insert size: 6.4 μm) *in vitro*[29]. Briefly, single cell suspensions of LM8G7 cells (2.5 x 10^4^) were prepared by detaching and resuspending the cells in DMEM containing 0.1 % BSA. Before the cells were added, the chambers were rehydrated for 2 h in an incubator at 37°C. The lower chambers were filled with DMEM containing 5 % FBS. To the upper chamber, LM8G7 or OVSAHO cells and compound **4g** in serum-free DMEM was added. After incubation for 24 h, cells that had passed through the BD BioCoat^TM^ chamber and remained attached to the opposite surface of the membrane are stained with Diff-Quick solution and counted in five random microscopic fields per filter. The % inhibition of the migration of LM8G7 or OVSAHO cells by compound **4g** was calculated.

### Real-time cell proliferation assay

The cell proliferation assay was done using the RT-CES^TM^ system (ACEA Biosciences, San Diego, CA). Briefly, LM8G7 cells (5x10^4^ cells) were added to ACEA 96X microtiter plates (e-plate^TM^) in 100 μl of medium [30]. In an experiment, cell monolayer was made and the various concentrations of compound **4g** (2 to 75 μM), in 150 μL of DMEM were individually added. The effect of the compounds on the proliferation of LM8G7 or OVSAHO cells was continuously monitored up to 48 hours for every 10 min. The proliferation was monitored for a period of 70 h and expressed as a cell index (quantitative measurement of cell proliferation) as per the manufacturer’s instructions. A cell index was plotted against time (for duplicate experiments). The IC_50_ values were calculated from concentration-response curves by a non-linear regression analysis using the GraphPad Prism (GraphPad Prism Software Inc., San Diego).

### MTT assay

The anti-proliferative effect of the compound **4g** on MCF-7, melphalan-resistant multiple myeloma (RPMI8226/LR5) MCF-10A or UV♀2 cells was determined by MTT dye uptake method as described previously [29]. Briefly, the cells were incubated in triplicate in a 96-well plate in the presence or absence of indicated concentration of compound **4g** in a final volume of 0.2 ml for different time intervals at 37°C. Thereafter, 20 μL MTT solution (5 mg/ml in PBS) was added to each well. After a 2 h incubation at 37°C, 0.1 ml lysis buffer (20 % SDS, 50 % dimethylformamide) was added; incubation was continued overnight at 37°C; and then the optical density (OD) at 570 nm was measured by Tecan plate reader.

### Annexin V Assay

One of the early indicators of apoptosis is the rapid translocation of the membrane phospholipid phosphatidylserine from the cell’s cytoplasmic interface to the extracellular surface and its accumulation there, producing a loss of membrane symmetry that can be detected using annexin V. Briefly, 1 x 10^6^ MCF-7 cells were pretreated with compound **4g** (30 μM) for various time points and then subjected to annexin V staining. Cells were washed, stained with FITC-conjugated anti-annexin V antibody, and then analyzed with a flow cytometer (BD FACS Calibur, BD Biosciences, US) [28].

## Authors' contributions

MPS and Basappa lead the medicinal chemistry efforts for the project and assisted in writing the manuscript. Basappa and MS conceived the project, established the in vitro assays, and participated in the experimental design and wrote the paper. SN, AC and DSP are responsible for experimental design of the study. GS was responsible for experimental design of the study, supervised KM, and FL, carried out the invasion assays and proliferation assays (MDA-MB-231, MCF-7 and RPMI 8266). KS and KSR designed the experiments, supervised MPS and Basappa. All authors read and approved the final manuscript.
